# The link between texting and motor vehicle collision frequency in the orthopaedic trauma population

**DOI:** 10.5249/jivr.v5i2.330

**Published:** 2013-06

**Authors:** Neil M. Issar, Rishin J. Kadakia, James M. Tsahakis, Zachary T. Yoneda, Manish K. Sethi, Hassan R. Mir, Kristin Archer, William T. Obremskey, Amir A. Jahangir

**Affiliations:** ^a^Vanderbilt Orthopaedic Institute, Center for Health Policy, Vanderbilt University Medical Center, Nashville, TN, USA.

**Keywords:** Texting, Driving, Trauma, Orthopaedic injury, Inattention, Motor vehicle collision

## Abstract

**Background:**

This study will evaluate whether or not texting frequency while driving and/or texting frequency in general are associated with an increased risk of incurring a motor vehicle collision (MVC) resulting in orthopaedic trauma injuries.

**Methods:**

All patients who presented to the Vanderbilt University Medical Center Orthopaedic Trauma Clinic were administered a questionnaire to determine background information, mean phone use, texting frequency, texting frequency while driving, and whether or not the injury was the result of an MVC in which the patient was driving.

**Results:**

237 questionnaires were collected. 60 were excluded due to incomplete date, leaving 57 questionnaires in the MVC group and 120 from patients with non-MVC injuries. Patients who sent more than 30 texts per week (“heavy texters”) were 2.22 times more likely to be involved in an MVC than those who texted less frequently. 84% of respondents claimed to never text while driving. Dividing the sample into subsets on the basis of age (25 years of age or below considered “young adult,” and above 25 years of age considered “adult”),young, heavy texters were 6.76 times more likely to be involved in an MVC than adult non-heavy texters (p = 0.000). Similarly, young adult, non-heavy texters were 6.65 (p = 0.005) times more likely to be involved in an MVC, and adult, heavy texters were 1.72 (p = 0.186) times more likely to be involved in an MVC.

**Conclusions:**

Patients injured in an MVC sent more text messages per week than non-MVC patients. Additionally, controlling for age demonstrated that young age and heavy general texting frequency combined had the highest increase in MVC risk, with the former being the variable of greatest effect.

## Introduction

With the advent of new technologies such as smaller, more mobile devices and phones capable of accessing email and the internet, cell phone use has skyrocketed in the past few decades. The ubiquity of smart phones and text messaging provides a significant source of driver distraction and inattentiveness, and many studies have attempted to quantitatively prove this hypothesis. A retrospective epidemiology study conducted in Quebec, Canada, in 2003 aimed to identify a link between cell phone use and motor vehicle collisions (MVC). After receiving over 36,000 questionnaires, the study concluded that cell phone users had a higher risk of incurring an MVC compared to non-users, with a dose-response relationship between the frequency of cell phone use and MVC risk.^1^ In addition, studies conducted by the Virginia Tech Transportation Institute (VTTI) suggested that text messaging, in particular, was associated with the highest risk of all cell phone-related tasks.^2^ Specifically, VTTI’s research demonstrated that text messaging while driving made a crash or near crash experience 23 times more likely than when driving without a phone, and that text messaging caused drivers to take their eyes off the road for an average of 4.6 seconds over a 6 second interval, which was the longest duration of time for any cell phone-related activity.^2^ Most recently, in a 2011 report on distracted driving, the Governors Highway Safety Association (GHSA) used surveys to conclude that about one eighth of drivers admitted to texting while driving, with younger drivers reporting texting while driving more frequently than older drivers.^3^ This latter result is important as teen drivers have the highest crash rate per mile driven of any age group, with crash rates declining with each year of increasing age but not reaching the lowest levels until after age 30.^4^

Thus, texting while driving seems to be reasonably prevalent and significantly dangerous; however, the lack of uniform prohibition in the US is disconcerting. Currently only thirty states, including Tennessee, have legislation banning texting while driving.^5^ Most studies, including the aforementioned one conducted by the VTTI, have demonstrated that cell phone use impairs driving performance by increasing driver inattentiveness, thereby significantly increasing the risk of MVC.^2,6^ Although this association appears intuitive, there are currently no studies linking texting while driving to actual trauma caused by MVCs. The majority of relevant research has been conducted under controlled simulated environments, and studies that evaluate actual risk of injury with texting behavior are rare. Moreover, while simulation studies allow the manipulation of independent variables in a randomized, controlled setting, there is disagreement about the applicability of their conclusions to actual driving. The aforementioned Quebec study ^1^identified a correlation between actual MVCs and cell phone use, but it simply looked at general phone use and did not address texting behavior or phone use while driving. Greater information concerning the connection between texting and actual MVC is needed. 

Some of the referenced retrospective studies did identify an association between cell phone use and MVCs, but various limitations prevented them from establishing a causal relationship. For example, the epidemiology study conducted in Canada was performed using cell phone bills, which did not provide information regarding cell phone use while driving, and did not include data regarding texting behavior. In addition, the realistic VTTI study found no statistical association between talking on the phone and MVC risk. A recent study conducted by the Highway Loss Data Institute determined that laws banning texting while driving have not decreased crash risk and in some cases the crash risk has paradoxically increased. ^7^ In addition, Goodwin et al. have studied the long-term effects of North Carolina’s 2006 law banning all cell phone use by drivers younger than 18. ^8^ Two years after the law took effect, cell phone use by teenage drivers in North Carolina was not significantly different than that of teenage drivers in South Carolina, where a cell phone restriction does not exist. A majority of teenagers interviewed were aware of the law but believed it was not enforced. ^8^ Furthermore, Braitman and McCartt used telephone surveys to conclude that while laws banning hand-held phone use seemed to discourage some drivers from using a phone while driving, laws banning texting in particular while driving had little effect on the reported frequency of texting while driving in any age group.^9^

These results and the limitations of the epidemiology studies have raised questions regarding the actual impact of cell phone use and/or texting on MVC risk. Accordingly, there is a need for more quantitative data supporting the association between cell phone use, texting, and MVCs. In addition, studies are needed to determine the association between cell phone use/texting while driving and resultant trauma sustained in MVCs. These studies would be critical in highlighting the healthcare costs that result from this dangerous behavior, and could help motivate legislation and community action that would save lives and healthcare dollars in the future. Accordingly, this study was designed to evaluate whether or not the frequency of texting while driving and/or general texting frequency is associated with an increased risk of incurring a motor vehicle collision.

## Methods

From October 2010 to March 2011, questionnaires were distributed to all trauma patients who presented to the Orthopedic Trauma Clinic at Vanderbilt University Medical Center in Nashville, TN. The questionnaire consisted of 13 brief questions divided into four categories – basic demographics, trauma details/medical information, automobile involvement in trauma, and cell phone usage. The motor vehicle portion consisted of three questions with the main goal of determining if the trauma was a result of an MVC and if the patient was driving at the time of the collision. The model of the vehicle was also collected in order to be able to differentiate between automobile and motorcycle collisions. The cell phone component contained three specific questions regarding phone use: how many hours per week do patients use their phone, how many texts per week do they send, and how many texts per week do they send while driving. For statistical purposes, hours spent talking on the phone was classified as “general phone use” and does not include texting.

Questionnaires were distributed to all new patients in the Orthopedic Trauma Clinic by the clinic nurses during their scheduled clinic visit. Patients who were unable to comprehend the questionnaire due to a language barrier or illiteracy were excluded from the study. Each questionnaire consisted of a brief disclaimer explaining the purpose of the study and ensuring patient confidentiality; patient participation was optional. Aside from the patient’s birth date, no other identifiable information was collected, and data was stored in a secured de-identified ¬dataset to further protect patient confidentiality. The study was approved by the Institutional Review Board of the Vanderbilt University School of Medicine. Collected questionnaires that were not complete were excluded from data analysis. The questionnaires that fit the inclusion criteria were grouped into two categories based on the specifics of the trauma. Patients who were involved in a MVC and were driving the vehicle at the time of the collision were assigned to Group A. All other patients were assigned to Group B. 237 questionnaires were collected. 60 questionnaires were excluded due to incomplete information such as demographic information, mechanism of injury, automobile information, and phone usage information. Out of the remaining 177 questionnaires, 57 fit the eligibility criteria to be assigned to Group A, while 120 were assigned to Group B. The average age of patients in Group A (MVC) was 38.0, and the age range was from 18 to 76. The average age of patients in Group B (non-MVC) was 44.4, and the age range was from 18 to 77.

Two logistic models were utilized, comparing the likelihood of being involved in a MVC for those that declared that they had sent more than 30 texts per week in general and those patients that had sent more than 30 texts while driving per week (“heavy texters”) to those who had sent less (“non-heavy texters”). The decision to use 30 texts as the cut-off for “heavy texters” was made to ensure sufficient separation of those individuals who very rarely or never engaged in texting behaviors from those who text more often. If a higher cut-off (such as 100 texts sent per week) had been chosen, the patients that only sent a few or no texts in a week would have been grouped with the majority of respondents, preventing a comparison of individuals with disparate texting behaviors. In addition, the logistic models were utilized to make the same comparison after separating the questionnaires by age – 25 years of age or younger (“young adult”) and 26 years of age or older (“adult”). This divided the sample into four subsets – young adult non-heavy texters, young adult heavy texters, adult non-heavy texters, and adult heavy texters. Finally, statistical analysis was performed using STATA 10.

## Results

Texting was found to be the cell phone activity associated with the greatest probability of being involved in a motor vehicle collision. In fact, the results indicated no association between heavy general phone use (deemed to be greater than four hours of talking on the phone per week) and the probability of being involved in a motor vehicle collision when compared to low or no phone use (p = 0.694) (Table 1). Logistic regression found no significant association between the risk of incurring an MVC and heavy general phone (greater than 4 hours per week) (p=.694). 

**Table 1:** General Phone Use Frequencies for MVC vs. non-MVC

Phone use (hours/week)	Group A (MVC)	Group B (non-MVC)
0 – 1	15 (26.3%)	32 (26.7%)
1 – 2	11 (19.3%)	24 (20.0%)
2 – 3	10 (17.5%)	16 (13.3%)
3 – 4	6 (10.5%)	13 (10.8%)
>4	15 (26.3%)	35 (29.2%)
Total	57	120

Patients who sent more than 30 texts per week were 2.22 times more likely to have presented to the Vanderbilt Orthopedic Trauma Clinic after being involved in an MVC in which they were driving (p = 0.015) compared with those who texted less frequently (Table 2). The frequencies of texting for MVC and non-MVC groups are displayed in Table 3. By contrast, the vast majority of patients (84%) claimed to never text while driving (Table 4). There were only two patients who reported themselves as sending more than 30 texts while driving per week (Table 4), and as such, an odds ratio for heavy texting while driving could not be calculated. Despite the lack of data, a linear regression showed that texting in general and texting while driving were indeed associated (p=0.000), though only 12.0% of the variance in texting in driving could be explained by texting in general (R^2^ = 0.1201). 

**Table 2:** Logistic Regression Results Indicating Likelihood of MVC for Heavy or High Texting Frequency

Variable	Odds Ratio	95% CI	p-value
30+ texts per week	2.22	(1.167 – 4.230)	0.015

**Table 3:** General Texting Frequencies for MVC vs. non-MVC

Texts Sent (per week)	Group A (MVC)	Group B (non-MVC)
0	13 (22.8%)	35 (29.2%)
1 – 10	8 (14.0%)	24 (20%)
11 – 20	5 (8.8%)	13 (10.8%)
21 – 30	1 (1.8%)	8 (6.7%)
>30	30 (52.6%)	40 (33.3%)
Total	57	120

**Table 4:** Texting While Driving Frequencies for MVC vs. non-MVC

Texts Sent (per week)	Group A (MVC)	Group B (non-MVC)
0	46 (80.7%)	104 (86.7%)
1 – 10	5 (8.8%)	12 (10.0%)
11 – 20	4 (7.0%)	1 (0.8%)
21 – 30	1 (1.8%)	2 (1.7%)
>30	1 (1.8%)	1 (0.8%)
Total	57	120

A subgroup analysis was conducted controlling for age, dividing the sample into four subsets – young adult non-heavy texters, young adult heavy texters, adult non-heavy texters, and adult heavy texters. Being a young adult alone increased the likelihood of incurring an MVC by 5.64 (p = 0.001), and heavy texting alone increased the same likelihood by 2.22 (p = 0.015) (Table 2). The subsets were compared against adult non-heavy texters, because this was the group with the lowest incidence of MVC. Young adult heavy texters were 6.76 times more likely to be involved in an MVC than adult non-heavy texters (p = 0.000), while young adult non-heavy texters and adult heavy texters were 6.65 (p = 0.005) and 1.72 (p = 0.186) times as likely, respectively (Table 5). However, it is important to note that large confidence intervals indicate greater levels of variance and therefore decreased accuracy and reliability of these odd ratios despite the presence of a significant association.

**Table 5:** Logistic Regression Results Indicating Likelihood of MVC for Age and Texting Frequency Combined

Subset	Odds Ratio	95% Confidence Interval	p-value
Adult, Heavy Texter	1.72	(0.771 – 3.822)	0.186
Young Adult, Non-Heavy Texter	6.65	(1.770 – 24.982)	0.005
Young Adult, Heavy Texter	6.76	(2.603 – 17.533)	0.000

However, an analysis of variance (ANOVA) demonstrated that a greater proportion of the elevated risk of incurring an MVC was due to age than frequency of texting. Attempts to explain this effect were made by repeating the analysis with different parameters. Treating age as a continuous variable showed that every additional year of age resulted in a 2.3% decreased risk of incurring an MVC (p = 0.066). Changing our comparison from “heavy texters” versus “non-heavy texters” to “texters” (those that declared that they had sent more than one text per week) versus “non-texters” (those that declared that they had sent no texts) showed that texting in general and texting while driving increased the likelihood of incurring an MVC by 1.45 (p = 0.259) and 1.61 (p = 0.216), respectively. 

## Discussion

The aim of this study was to investigate whether or not the frequency of texting while driving and/or general texting frequency was associated with an increased risk of incurring an MVC. A number of associations became clear from the data. First, as the VTTI research demonstrated, texting was found to be the cell phone activity associated with the greatest probability of being involved in a motor vehicle collision.^2^ In fact, our results indicated no association between heavy general phone use and increased probability of being involved in an MVC when compared to low or no phone use. Conversely, patients who were heavy texters (sent more than 30 texts per week) were 2.22 times more likely to be involved in an MVC than those who texted less frequently. Accordingly, the specific act of manually manipulating a cell phone (as in receiving or sending a text message) may be a particularly significant source of driver inattention contributing to the increased incidence of motor vehicle collisions. These results have been supported in previous studies including the VTTI study, as well as an 18-month-long simulator study from the University of Utah, which showed an eight times greater motor vehicle collision risk when texting than when not texting among college students. ^10^

Our results do not allow us to conclude that heavy texting specifically while driving is associated with an increased risk of being involved in a MVC. It is noteworthy that a mere two patients reported themselves as sending more than 30 texts per week while driving. In fact, the vast majority of patients (84.36%) claimed to never text and drive; however, patients are likely hesitant to admit to texting while driving, as Tennessee is one of the states in which texting while driving is legally prohibited. Various studies have shown texting frequencies, both in general and while driving, to be increasing in past years independent of texting bans or legislation, particularly among young drivers.^11,12^In addition, a linear regression showed that texting in general and texting while driving were associated with statistical significance. Consequently, despite a low R-squared, we may assume some correlation between general texting frequency and frequency of texting while driving among our sample, lending clinical significance to the aforementioned association between general texting frequency and likelihood of being involved in an MVC. 

We must also recognize that age has a considerable demonstrated impact on the likelihood of incurring an MVC.^13^Accordingly, it is prudent to divide our sample into subsets and analyze the effect of both of these variables. The distinction between “young adult” and “adult” drivers was set at 25, because this is the age at which car insurance rates usually drop, indicating that crash rates go down at this age. Young adult heavy texters were most at risk for incurring an MVC, with a 6.76 times increase in probability as compared to adult non-heavy texters. Since age and heavy texting alone do not increase the likelihood of incurring an MVC to such an extent, we can conclude that both age and a high frequency of texting are correlated with the likelihood of incurring an MVC and that these variables are not independent of each other. However, a two-way ANOVA demonstrated that age was a more significant contributing factor to the elevated risk of incurring an MVC than frequency of texting. Treating age as a continuous variable (instead of “young adult” versus “adult”) did not produce useful information, as the risk of incurring an MVC decreased negligibly with increasing age, and car insurance companies almost always treat age as a discrete variable when determining insurance rates. In addition, changing the comparison from “heavy texters” versus “non-heavy texters” to “texters” versus “non-texters” in an effort to explain this contributory effect produced statistically insignificant results. Thus, despite this study’s initial aim of determining whether or not an association exists between texting while driving and/or general texting frequency and an increased risk of incurring an MVC, it appears the more statistically significant association exists between age and MVC risk. 

There are several limitations of the study. First, the study’s analysis and conclusions could be strengthened by increasing the sample size, as 237 questionnaires may be considered too few to determine accurate MVC probabilities. Second, the determination of our cut-offs for statistical analysis also has certain implications. For example, we chose to use 30 texts per week as the cut-off to define “heavy texters”, which may be considered lower than the definition of “heavy texters” used by others. While our subjectively-selected cut-off facilitated a separation between those who rarely engaged in texting behaviors and those who texted more frequently, one must exercise caution when applying our results to situations implying variable cut-offs. Third, there may be recall or memory bias, as we have no way of determining the accuracy of patient’s recollection of their average phone use and texting habits. There may also be some response bias, as patients may presume they are being tested to determine adherence to Tennessee’s laws against texting while driving, thereby pushing more patients to answer that they do not text and drive at all. Fourth, we were unable to separate the effects of age and cell phone use on MVC risk. Most notably, while this study identified an association between general texting frequency and MVCs, the study did not identify a conclusive link between texting while driving and MVCs in this sample population. Over 84% of the patients claimed to never text while driving and there was no way to determine if patients were texting at the time of their accident. This study and other previously conducted retrospective studies identify the need for a better tool to quantitatively measure texting while driving habits, specifically in the time immediately preceding an MVC. For example, a structured interview could be a better way to overcome the response bias that may prevent patients from truthfully answering questions. Assurance that results will be kept confidential can be more powerful when told in person as opposed to a written disclaimer at the end of a survey tool. Regardless, until researchers are able to develop methods to overcome this barrier, it will be difficult to make a claim of causality between cell phone use and MVCs. This lack of established causation is perhaps one of the reasons why more comprehensive legislation against texting while driving does not exist today.

This study is one of the first to examine the association between texting behavior and increased MVC frequency using actual hospital patient data. This association is crucial to determining the effect of texting while driving on the health care costs incurred by physicians and hospitals. A conclusively-demonstrated association could be the first step to invoking national legislation regarding texting while driving. The US Department of Transportation issued regulatory guidance in January 2010 prohibiting text messaging by commercial motor vehicle drivers,^14^ but studies on the link between trauma and texting from other large healthcare institutions are needed to transform regulatory guidance into legislative restriction. Furthermore, the decide to drive national public service campaign highlighting the dangers of texting while driving, led jointly by the American Academy of Orthopaedic Surgeons (AAOS) and the Orthopaedic Trauma Association (OTA), would benefit from the results of this study and similar ones. Legislation alone may not be sufficient to curb the dangerous behavior of texting while driving. As evidenced by the GHSA report,^3^ laws banning hand-held cell phone use while driving tend to cause an initial sharp decrease in cell phone use followed by a gradual increase. Regardless of the method adopted to limit texting while driving, it is most important to target young novice drivers who are already at higher crash risk (as demonstrated by the results of this study) and thereby more likely to suffer serious consequences from distracting behaviors like texting while driving.

## Conclusion

Patients who sustained orthopaedic injuries as a result of a motor vehicle collision were younger and sent more text messages per week than those patients who were older and/or injured by a cause other than a motor vehicle collision. Both factors (young age and high frequency of texting) combined demonstrated the greatest increase in risk of being involved in an MVC. Texting is a particularly hazardous form of driver inattention that increases the likelihood of being involved in a motor vehicle collision, even compared to other forms of phone use, and that awareness campaigns and legislation regarding the issue should target young drivers as the population group most at risk.
